# RE: Comments on cIMPACT‐NOW Update 11

**DOI:** 10.1111/bpa.70061

**Published:** 2026-01-12

**Authors:** Henning Leske, Richard Doughty, Pitt Niehusmann

**Affiliations:** ^1^ Department of Pathology Oslo University Hospital, Rikshospitalet Oslo Norway

**Keywords:** cIMPACT‐NOW, diffuse paediatric‐type high‐grade glioma, glioblastoma, IDH‐wildtype, histone, posterior fossa ependymoma, WHO classification

We read with great interest cIMPACT‐NOW Update 11, in which the consortium provides diagnostic recommendations for glioblastoma, IDH‐wildtype (GBM); diffuse paediatric‐type high‐grade glioma, H3‐wildtype and IDH‐wildtype (pHGG); and posterior fossa ependymal tumors. The authors are to be commended for addressing unresolved challenges identified since the release of the 5th edition of the WHO Classification of Tumours of the Central Nervous System (WHO CNS5, 2021). While the update offers valuable guidance, we would like to comment on several aspects that would benefit from clarification, closer alignment with WHO CNS5, and refined wording to avoid diagnostic ambiguity in routine practice.

## 1 GLIOBLASTOMA, IDH‐WILDTYPE (GBM)

WHO CNS5 specifies an age of “≥55 years” in the absence of *IDH1* p.R132H immunoreactivity, classic GBM histology, non‐midline location, and no history of a pre‐existing lower‐grade glioma as sufficient for a GBM diagnosis. The repeated use of “>55 years” in Update 11—implying a shift from ≥55 to ≥56 years—appears to be a typographical inconsistency, which should be confirmed by the authors [1].

WHO CNS5 also recommends that ATRX‐negative tumors should undergo additional molecular testing to exclude *IDH1*/*2* and/or H3 alterations, regardless of age. Update 11 highlights H3 p.K28me3 immunostaining as a practical method to exclude diffuse midline glioma, H3 K27‐altered (DMG)—useful not only for H3 p.K28M‐mutant cases, as mentioned by the authors, but also for those driven by *EZHIP* overexpression or *EGFR* sequence alterations, as retained nuclear expression virtually excludes DMG.

The committee's recommendation that *TERT*‐promoter mutations in IDH‐wildtype astrocytic gliomas with low‐grade histology should not, in isolation, define GBM is prudent to prevent overtreatment in a subset of cases, particularly given recently characterized tumors such as adult‐type diffuse high‐grade glioma, IDH‐wildtype, subtype F (HGG‐F) with less aggressive clinical behavior. However, the suggestion that some tumors with “higher‐grade features that do not meet histologic criteria for glioblastoma” be diagnosed descriptively (not elsewhere classified, NEC) introduces ambiguity. It remains unclear whether such cases with absence of necrosis and vascular proliferation—despite high mitotic activity and isolated *TERT*‐promoter mutation—do not justify a GBM diagnosis.

The statement that isolated *EGFR* amplification or +7/−10 signature alone is not sufficient for a GBM diagnosis in patients <40 years is reasonable, but somewhat rigid. It should be stressed that these are expert recommendations intended to guide pathological practice. However, since the authors also note that diffuse paediatric‐type high‐grade glioma, H3‐wildtype and IDH‐wildtype (pHGG) as one of the major differential diagnoses can occur “in adults up to 70 years of age,” this rule of thumb may be adapted to local resources and should not refrain pathologists in suspicious cases from performing additional analyses in older patients.

In the summary table at the end of the GBM section, the designation “GBM, IDH‐wildtype and H3‐wildtype” is potentially misleading. While the term H3‐wildtype excludes the relevant differential diagnosis of diffuse hemispheric glioma, H3 G34‐mutant, the retained nuclear H3 p.K28me3 immunoreactivity—not simply “H3‐wildtype”—is the relevant criterion that excludes DMG. Moreover, the phrasing “*TERT*‐promoter mutation (not as solitary defining factor in histologically low‐grade tumors)” conflicts with earlier references to “higher‐grade features” and requires clarification.

Update 11 also discusses low‐confidence matches in DNA methylation analysis and states that a high‐confidence match to the GBM, IDH‐wildtype methylation family is sufficient, and that “score(s) for the methylation subclass(es) do not affect the WHO CNS5 integrated diagnosis nor the therapeutic management.” However, the terminology and interpretation around classifier hierarchy are somewhat unclear. Methylation classifier outputs are structured hierarchically as superfamily > family > class > subclass. As cIMPACT‐NOW Update 9 discusses, there are inconsistencies across entities regarding the diagnostic sufficiency of different hierarchy levels [2]. While Update 11 refers in GBM diagnostics to subclass scores as dispensable, in fact it is not only the subclass but also an insufficient score of the methylation class that may be disregarded in the final diagnostic integration, provided the methylation family match is of high confidence. This distinction should be clearly stated, as it has direct implications for diagnostic decisions and reporting consistency.

As a minor detail, WHO CNS5 lists only giant cell GBM, gliosarcoma, and epithelioid GBM under “subtypes,” but also discusses small cell GBM, GBM with primitive neuronal component, granular cell GBM, and heavily lipidized GBM [1]. Since these variants are defined based on histological features, they should likewise be acknowledged in Update 11 for completeness and consistency.

## 2 DIFFUSE PAEDIATRIC‐TYPE HIGH‐GRADE GLIOMA, H3‐WILDTYPE AND IDH‐WILDTYPE (pHGG)

We welcome the effort to provide guidance for this diagnostically challenging entity. In the past, pHGG has frequently been identified only through methylation profiling, which distinguishes it from GBM despite overlapping histological features and molecular alterations such as +7/−10, *EGFR* or *PDGFRA* amplification, and *TERT*‐promoter mutations. Update 11 appropriately acknowledges that DNA methylation profiling is currently the only reliable approach for pHGG classification.

Update 11 notes that in IDH‐wildtype diffuse high‐grade glioma patients below 25 years of age, *PDGFRA* or *EGFR* alterations may suffice for pHGG diagnosis if methylation profiling is unavailable. While this may be valid in *MYCN*‐amplified cases, *PDGFRA* and *EGFR* alterations, as the authors also point out, are not specific and occur in GBM. In such contexts, a descriptive diagnosis such as diffuse high‐grade glioma, *MYCN*‐amplified with a “not otherwise specified (NOS)” amendment is preferable to a definitive WHO type assignment as pHGG, *MYCN*‐amplified, NOS to acknowledge uncertainty and avoid misclassification.

Furthermore, the statement that methylation profiling alone reliably distinguishes pHGG subtypes—despite detection of molecular alterations such as *PDGFRA*, *EGFR*, or *MYCN* amplification by molecular testing—may overstate current evidence. The clinical and therapeutic relevance of these subtypes remains insufficiently understood and requires further validation.

While the WHO CNS5‐conform entity name matches the methylation family provided by methylation classifiers, it remains unclear why a match with the methylation class is needed for a diagnosis of pHGG, particularly since no other molecular alterations are claimed to be sufficient for such a diagnosis. This requirement risks complicating the diagnostics of pHGG.

## 3 POSTERIOR FOSSA EPENDYMAL TUMORS

The discussion of posterior fossa ependymomas addresses an important diagnostic challenge. WHO CNS5 mandates methylation profiling in ependymal tumors to distinguish between posterior fossa ependymoma (group B) and subependymoma. However, as pointed out by the authors, tumors with classic ependymoma morphology but harboring a *TERT*‐promoter mutation and/or chromosome 6 loss were recently described and are currently assigned to the posterior fossa subependymoma (PFSE) methylation class. The progression‐free survival of these tumors aligns more closely with CNS WHO grade 2 ependymomas rather than the favorable CNS WHO grade 1 outcome expected of subependymomas.

It is therefore puzzling that Update 11 proposes reliance on PFSE methylation class assignment in histologically pure ependymomas by suggesting the diagnosis of a “posterior fossa ependymal tumor with *TERT*‐promoter mutation and/or chromosome 6 loss and with PFSE methylation class assignment, NEC.” Reliance on PFSE in the classification of such cases should be reconsidered until a revised framework is provided in WHO CNS6.

Finally, we regret the authors' decision to abandon Human Genome Variation Society (HGVS)–compliant histone nomenclature “for the sake of brevity” throughout the manuscript. In doing so, they incorrectly cite tables 3 and 4, where changes are neither presented according to HGVS guidelines nor clearly highlighted as such. Standardized terminology following HGVS recommendations, as adopted by WHO CNS5, ensures clarity in both diagnostics and research reporting. Its omission risks reintroducing the very inconsistencies that HGVS‐based nomenclature was designed to resolve [3, 4].

## 4 CONCLUSION

cIMPACT‐NOW Update 11 provides important interim guidance for CNS tumor classification and addresses areas of significant diagnostic uncertainty. However, several areas warrant refinement to improve clarity, consistency, and alignment with WHO CNS5:

- Clarification of the age cut‐off discrepancy for GBM diagnosis in *IDH1* p.R132H‐negative diffuse gliomas with GBM features, non‐midline location, and without a history of a pre‐existing lower‐grade glioma.
- Clarification whether a descriptive diagnosis in IDH‐wildtype diffuse gliomas with *TERT*‐promoter mutation as the solitary finding in the absence of classic GBM histology is recommended only in low‐grade astrocytic gliomas or also in cases with higher‐grade histological features.
- Clarification of the sufficiency of methylation family (vs. class) match in the diagnosis of GBM and pHGG.
- Reassessment whether *EGFR*/*PDGFRA*‐alterations suffice for pHGG, NOS diagnosis in patients <25 years without methylation profiling.
- Critical evaluation of reliance on PFSE methylation class assignment for “posterior fossa ependymal tumor with *TERT*‐promoter mutation and/or chromosome 6 loss.”
- Reinstatement of HGVS‐compliant nomenclature to promote international consistency and reproducibility.

Addressing these issues would strengthen diagnostic precision, enhance consistency with WHO CNS5, and ultimately support improved diagnostics and patient care.

## AUTHOR CONTRIBUTIONS

All authors of this manuscript participated in discussions, contributed to writing, and approved the manuscript.

## CONFLICT OF INTEREST STATEMENT

The authors declare no conflicts of interest.

## Data Availability

The correspondence letter is related to a previously published work in *Brain Pathology* (https://doi.org/10.1111/bpa.70035). The data provided are based on the references listed within the manuscript and personal experience.
